# Paediatric hospitalizations over three waves of COVID-19 (February 2020 to May 2021) in Italy: determinants and rates

**DOI:** 10.7717/peerj.15492

**Published:** 2023-06-23

**Authors:** Manuela Martella, Alberto Peano, Gianfranco Politano, Roberta Onorati, Maria Michela Gianino

**Affiliations:** 1 Department of Public Health Sciences and Pediatrics, University of Turin, Turin, Piedmont, Italy; 2 Department of Control and Computer Engineering, University of Turin, Turin, Piedmont, Italy; 3 Epidemiology Unit, Local Health Authority TO3, Grugliasco (Turin), Piedmont, Italy

**Keywords:** Hospitalization, Children, Adolescent, COVID-19, Determinants

## Abstract

**Background:**

After a pneumonia outbreak in late 2019 in China, a new virus related to the Coronaviridae strain, called Severe Acute Respiratory Syndrome-Coronavirus-2 (SARS-CoV-2), was identified as the pathogen of an emerging disease known as COronaVIrus Disease 19 (COVID-19). Preliminary evidence outlines a higher prevalence in adults and lower susceptibility in children. However, recent epidemiologic research highlighted that transmissibility and susceptibility among children and adolescents become higher due to new virus variants. Infections among youth arises with mainly respiratory and gastrointestinal symptoms and malaise. Nevertheless, critical illness affects new-borns and fragile children, requiring hospitalization and possibly intensive care support. Aim of this study was to examine the impact of COVID-19 pandemic on hospital admissions among children and adolescents aged 0 to 17 years over three waves of COVID-19 (from February 2020 to May 2021) in Piedmont, a large Italian region, and to investigate the possible determinants of hospitalizations.

**Methods:**

A meta-analysis for risk assessment was performed over three waves of COVID-19 (from February 2020 to May 2021). Data were extracted from the official Italian National Information System and ISTAT.

**Results:**

Overall, 442 paediatric patients were enrolled and admissions concerned mostly the age group 0–4 years (60.2%). Trends of hospitalization showed a slight increase of paediatric admissions already in March 2020 and a rise during second and third waves (November 2020, March 2021). Paediatric age-grouped hospitalizations (0–4; 12–17; 5–11) reproduced an analogous trend. The children and adolescent hospitalization rate appeared lower than overall population with a moderate slope of increase in comparison with population slope. Monthly hospitalization rate (per 100,000) of children and adolescents aged 0–17 years reproduced the increasing trend of hospitalization numbers. This trend was influenced, in particular, by the trend of hospitalization rates for children aged 0–4 years. The meta-analysis for risk assessment showed a decreased likelihood of rescue of hospitalizations in female, 5–11 and 12–17 age groups. Conversely, the meta-analysis showed a positive association between foreign nationality and hospitalizations.

**Conclusions:**

Our results show a comparable trend of paediatric hospital admissions for COVID-19 and of the entire population hospitalizations over three waves. COVID-19 hospital admissions increase with a bimodal age distribution and the most admissions are among patients aged ≤4 or 5–11 years. Significant predictive factors of hospitalization are identified.

## Introduction

After a pneumonia outbreak in late 2019 in the region of Wuhan (China), a new virus related to the Coronaviridae strain, called Severe Acute Respiratory Syndrome-Coronavirus-2 (SARS-CoV-2), was identified as the pathogen of an emerging disease known as COronaVIrus Disease 19 (COVID-19) (Wu et al., 2020). On 11th March 2020, the World Health Organization (WHO) declared worldwide a pandemic health emergency (WHO Director-General, 2020), and 11,538 cases and 619 deaths had already been reported in Italy on that day. The overall mortality rate was 5.4%, affecting exclusively the older age groups. Among children and adolescents (0–18) just 1.2% of cases and no deaths were registered. Most cases occurred in Northern Italy and Piedmont was the fifth region with the highest prevalence (Epicentro—Istituto Superiore di Sanità, 2020).

Preliminary evidence outlines a higher prevalence (*i.e.*, sero-prevalence) in adults and lower susceptibility to SARS-CoV-2 especially in children. According to several studies carried out during the first outbreak in 2020, chain of transmission of SARS-CoV-2 from parents to children namely from household contacts seem to be predominant. Although children are not likely to be super-spreader, as usually happens for viral respiratory epidemics, the secondary role of youth in viral transmission remains vague (Viner et al., 2021; Armann et al., 2020). The quick viral spread and its rapid genomic mutation have allowed the diffusion of several variants, like Delta variant (B.1.617.2), whose higher transmission rates and shorter average incubation period are probably the concurrent causes of the rising infection within younger age groups. Additionally, the increasing vaccination coverage in older adults has driven disease transmission further among children and adolescents (Howard-Jones et al., 2022).

The latest data showed that a minority of children requires hospital admission or intensive care admission; nevertheless, this may change whether new variants emerge. Accordingly, recent epidemiologic research highlighted that transmissibility and susceptibility among children and adolescents become higher due to new virus variants (Chen et al., 2022; Zimmermann et al., 2022).

SARS-CoV-2 infections among youth arises similarly to other common viral infections, with a wide range of respiratory and gastrointestinal symptoms and malaise. Critical illness affects mostly new-borns, and children and adolescents with underlying medical conditions (*i.e.*, obesity, diabetes, asthma, cancer). The severity of disease is associated with high rate of hospitalization and possibly Intensive Care Unit (ICU) admission due to serious pulmonary and extra-pulmonary symptoms. Complications are rare, especially in healthy children and complications, which require intensive care support, consist in acute lung injury that usually occurs 1–2 weeks after early manifestation, whereas cardiovascular, gastrointestinal and neurological involvement are peculiar for MIS-C and follow the symptom onset in 4–6 weeks (Howard-Jones et al., 2022; Hoste, Van Paemel & Haerynck, 2021; Esposito et al., 2021).

Several studies have been carried out on hospitalizations associated with COVID-19 among children and adolescents aged 0–17 years in different pandemic periods and multiple countries (Siegel et al., 2021; Delahoy, 2021).

However, little is known about the trend of children admissions during three waves of COVID-19 in the Italian health system and at our knowledge, there are not studies regarding the determinants of children admissions.

The aim of this study was to examine the impact of COVID-19 pandemic on hospital admissions among children and adolescents aged 0 to 17 years in Piedmont during the timespan February 2020-May 2021 and to investigate the determinants of hospitalizations.

## Materials and Methods

A cross-sectional time series analysis was carried out over a period of 16 months, from February 2020 to May 2021, in the Piedmont Region, which is the second largest region in Italy, with a population of more than 4 million inhabitants over an area of 25,387 km^2^ (ISTAT, 2022). The period from February 2020 to May 2021 covers three waves of COVID-19.

Selection of hospital admissions with COVID-19 diagnosis code in Piedmont among people aged from 0 to 17 years and extraction of pertaining data were performed.

The following variables were collected: sex, age (grouped as 0–4, 5–11, 12–17), nationality (Italy, EU countries, Extra-EU countries); date of hospital admission (month and year), length of stay (LOS), provenience of patient (Emergency Department—ED, general practitioner, other hospital, wards of the same hospital, others) and destination after discharge (other hospital, home, died). The “wards of the same hospital” referred to intra-hospital transfers from neonatology unit after birth or after scheduled hospitalizations in other wards.

These data were collected from patients’ hospital discharge database (SDO database). Data were extracted from the Health Information System of the Piedmont Region, in accordance with regulations of protection of personal data in force; exclusively delegates belonging to the Regional Epidemiology Network are in charge to download and process data. The Regional Epidemiology Network provides for digital collection of health data coming from different sources through such Health Information System, in support to epidemiological surveillance activities and assessment of population health status and evaluation of needs of the community. The Unit of Epidemiology-regional Health Service of the Local Health Board TO3 is a node of the network and stipulated a formal agreement between the units involved in this study, also for research purposes.

The platform COVID-Stat (National Institute for Nuclear Physics, 2022) from the National Institute for Nuclear Physics (INFN) provided daily data of hospitalizations reported in Italy, from end of January 2020 until now.

Aggregate data, for the entire Italian population of all ages, and stratified ones, for region and for age, were downloaded from the platform as Comma-Separated Values (CSV) files to create tables and graphics.

Data analysis focused on computing monthly hospitalizations among overall population resident in Piedmont, monthly hospitalizations for people aged 0–17 and related age-groups (0–4; 5–11 and 12–17) resident in Piedmont, as well. Additionally, hospitalization rate was calculated as number of monthly (overall or age specific) hospitalizations divided by yearly (overall or age specific) population in Piedmont per 100,000. Following trends were compared: overall and age-grouped number of paediatric hospitalizations, overall and age-grouped paediatric hospitalization rates, as well. Further, a comparison between number of hospitalizations and hospitalization rates among general population and among people aged 0–17 was evaluated.

Data source of the census of population updated to the 1st January 2020 and to the 1st January 2021, including nationality, was the official platform of the Italian Institute of Statistics (ISTAT) (ISTAT, 2021a, 2021b).

### Statistical analysis

A meta-analysis for risk assessment was performed to assess the odds of hospitalizations across selected independent factors, namely sex, nationality (Italian and Foreign-EU/extra-EU) and age groups, over the 16-month study period (European Centre for Disease Prevention and Control, 2021).

The R framework (R Core Team, 2013) was used to perform all analyses and the significance level was set at *p* < 0.05 for all analyses.

## Results

### Descriptive analysis of the sample

Data from 442 paediatric patients recorded under COVID-19 diagnosis code were collected during the period February 2020 and May 2021. Most of patients aged from 0 to 4 years (60.2%), whereas 20.4% and 19.4% belonged to the age groups 12–17 and 5–11 years, respectively, the mean age was 5.03 (SD 5.74).

Patients were predominantly males (58.6%) and 77.1% of patients were Italian. More than half of hospitalizations occurred during 2020 (55.6%). The mean LOS was 5.3 (SD 5.4) days. Admission of 280 patients (63.3%) occurred through the ED. Provenience from the other hospitals represented the 16.7% of admissions. In addition, 7% were intra-hospital transfers (from neonatology unit after birth or by scheduled hospitalization), whereas 7.2% of patients got access through request from general practitioner (GP) or other specialist. Places of discharge were mostly home (92.3%) and only 7% was discharged to other healthcare setting. Finally, three patients (0.7%) died (Table 1).

**Table 1:** Patient’s characteristics.

	Overall sample		
	*N* (%)		
Age group			
0–4	266 (60.2)		
5–11	86 (19.4)		
12–17	90 (20.4)		
Gender			
Male	259 (58.6)		
Female	183 (41.4)		
Nationality			
Italian	341 (77.1)		
EU	32 (7.2)		
Extra-EU	69 (15.6)		
Overall hospital admission per year			
2020	251 (56.8)		
2021	191 (43.2)		
Provenience of patients			
ED	280 (63.3)		
Other hospital	75 (16.7)		
GP	32 (7.2)		
Other ward	31 (7.0)		
Other	24 (5.4)		
Destination after discharge			
Other hospital	31 (7.0)		
Home	408 (92.3)		
Deceased	3 (0.7)		
	Mean (SD)	Median	IQR
Age (years)	5.03 (5.74)	2	0–10
Lenght of stay (days)	5.3 (5.4)	4	2–6

**Note:**

N: total sample size; SD, standard deviation; IQR, Interquartile Range; ED, emergency department; GP, general practitioner.

**Figure 1:**
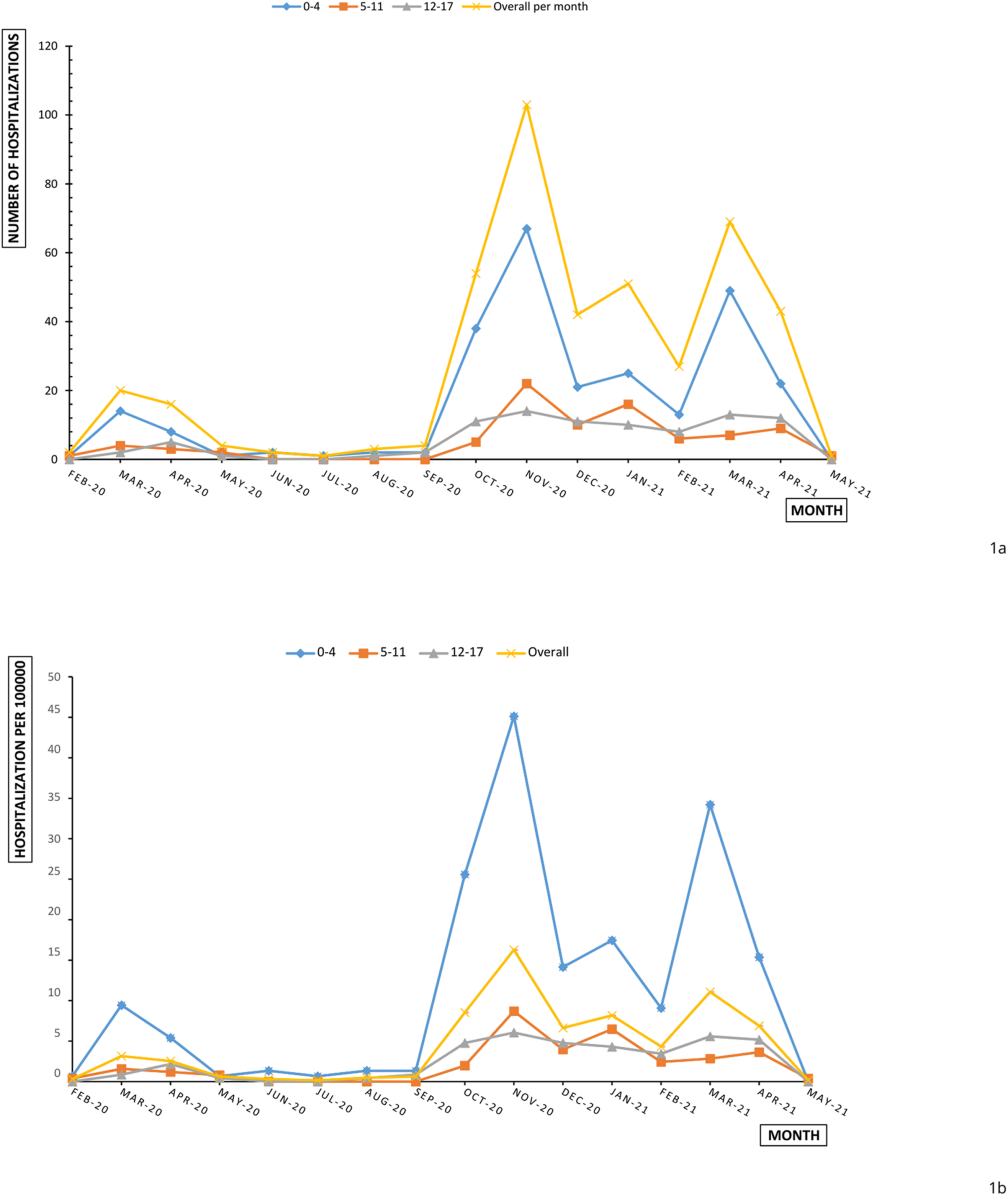
Overall and age-specific monthly paediatric hospitalizations (A) and hospitalization rates (B) per 100,000 in Piedmont during February 2020 and May 2021.

Figure 1A shows a slight increase of paediatric admissions already in March 2020; afterwards, hospitalizations settled around very few events until September 2020. In the second half of monitoring period–second and third wave—a dramatic rise of cases was reported in November 2020 and in March 2021.

**Figure 2:**
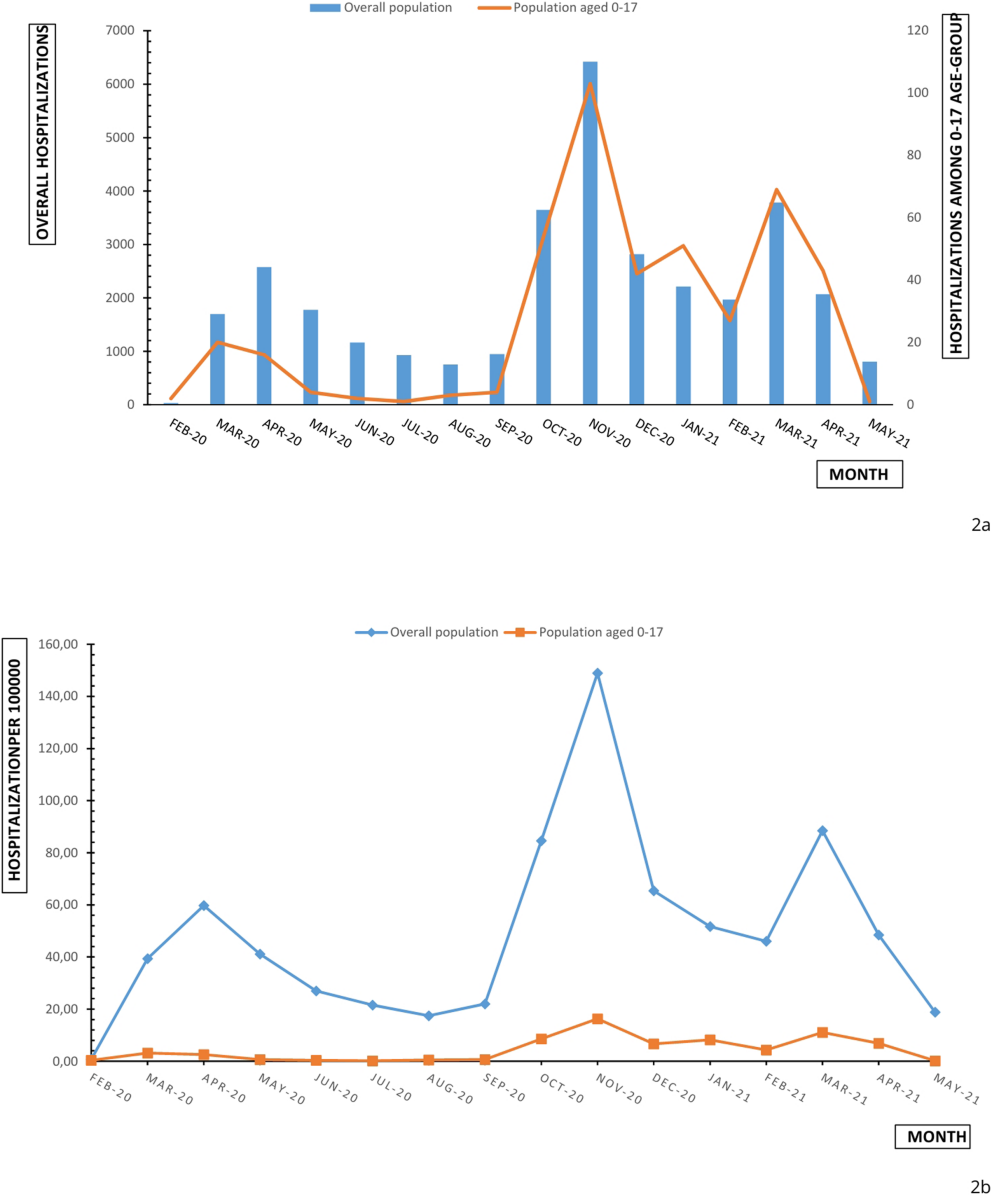
Monthly overall and age-specific (0–17) hospitalizations (A) and hospitalization rate (B) in Piedmont during February 2020 and May 2021.

Comparing paediatric data with the admission data of all Piedmont population, spikes match and a similar tendency is outlined (Fig. 2A). Children and adolescent account for 0.1% to 2.3% of reported COVID admissions across Piedmont region. The percentage increases from the first wave (0.7%) to the second wave (1.5%) to the third wave (1.8%). Paediatric age-grouped hospitalizations (0–4; 12–17; 5–11) reproduced an analogous trend.

Monthly hospitalization rate (per 100,000) of children and adolescents aged 0–17 years reproduced the increasing trend of hospitalization numbers (Fig. 2A). This trend was influenced, in particular, by the trend of hospitalization rates for children aged 0–4 years. Lower rates were observed for adolescents (12–17 years), for whom hospital admission incidence remained stable under 10 hospitalizations for 100,000 between October 2020 and April 2021 (Fig. 1B). The cumulative hospitalization rates over the studied period were 181.83, 34.37 and 38.8 for 0–4, 5–11 and 12–17 respectively. The children and adolescent hospitalization rate appeared lower than overall population with a moderate slope of increase in comparison with population slope (Fig. 2B).

The meta-analysis for risk assessment, reported in Table 2, showed a decreased likelihood of rescue of hospitalizations in female (Odds Ratio—OR 0.75, *p*-value < 0.001), 5–11 and 12–17 age groups (OR 0.19 and OR 0.22, *p*-value < 0.001 respectively). Conversely, the meta-analysis showed a positive association between foreign nationality and hospitalizations (OR 1.83, *p*-value < 0.001).

**Table 2:** Odds Ratios and 95% confidence interval (95% CI) of hospitalizations across selected independent factors (sex, nationality and age) over the 16-month study period.

	OR	95% CI		*p*-value	
Age group					
0–4	1				
5–11	0.19	[0.15–0.24]		*p* < 0.001	
12–17	0.22	[0.17–0.28]			
Gender					
Male	1				
Female	0.75	[0.62–0.9]		*p* < 0.001	
Nationality					
Italian	1				
Foreign (EU and Extra-UE)	1.83	[1.47–2.28]		*p* < 0.001	

## Discussion

The aim of this study was to examine the impact of COVID-19 pandemic on hospital admissions among children and adolescents aged 0 to 17 years in Piedmont during the timespan February 2020-May 2021 and to investigate the determinants of hospitalizations.

The results highlighted that, during the observed period, absolute number of admissions for COVID-19 for children and adolescents in Piedmont showed a fluctuating trend with peaks at the three waves, and consistently similar to distribution reported among all ages in the whole region during the same timeframe.

The percentage of paediatric admissions out of the total hospitalizations of the whole population increased from the first to third wave. One possible explanation could be the beginning of the COVID-19 vaccines administration (May 2021 for children aged 12 years onward and November 2021 for children aged from 5 to 11 years) that involved the elderly population leaving the younger population with no protection.

Trend of hospitalizations and admission rates respectively showed a gradual decrease after March 2020, as a consequence of implementation of containment restrictions and drastic preventive actions, such as quarantine, school closure and social restriction (Camera dei deputati-Documentazione parlamentare, 2022). From April 2020 to July 2020, age-specific (0–17) hospitalization rate decreased from 2.53 to 0.16 admission per 100,000, remaining under one admission per 100,000 till October 2020. These data reflect the flattening of the curve of the COVID-19 case notification rate reported from the European surveillance system (TESSy) for all age groups (European Centre for Disease Prevention and Control, 2021).

The increases in COVID-19 hospital admissions found in this study occurred for all assessed paediatric age groups during the three waves, with most admissions among patients aged ≤4 and 5–11 years. This bimodal age distribution is only partially consistent with other published data (Siegel et al., 2021; Kainth et al., 2020) that showed a different bimodal age distribution: 0–4 and 12–17. The strong presence of hospitalizations for children of 0–4 years is however, a recurring result in the literature. To confirm the mentioned studies, a national survey conducted by the German Society for Paediatric Infectious Diseases highlighted a high proportion of infants admissions: among 128 inpatients, 37% of them were infants and 29% were 1 to 5 years old (Armann et al., 2020).

Hospitalization rates among persons aged 0–17 showed increasingly lower values than that of the whole population and showed a flatter trend throughout the analysed period.

It is possible that the lower seroprevalence in younger than 18 years reported in several studies, in comparison with adults, may have resulted in lower incidence of hospitalization (Viner et al., 2021; Pollán et al., 2020; Stringhini et al., 2020).

Admissions rates concerned mostly the age group 0–4 years (60.2%) with a cumulative rate of 181.83/100,000. This result is not surprising, because it is in line with that of other countries. Center for Disease Control and Prevention (CDC) in US reported similar results about high hospitalization rates among children 0–4 years by depicting increasing cumulative COVID-19-associated hospitalizations for 100,000 children and adolescents between March 2020 and August 2021 (Delahoy et al., 2022).

Due to literature reported that those younger than 10 to 14 years are less susceptible to SARS-CoV-2 infection than those 20 years and older, resulting in lower prevalence (Viner et al., 2021), it is not easy to find an explanation to the result that the hospitalization rate was higher for the 0–4 range. A suggestion can be that parents of younger children seek for medical help more often than older children and adolescents. The infants’ vulnerability to severe COVID-19 is another possible explanation to this finding (Raba et al., 2020). These explanations also support another important result of the study according to which the age groups (5–11 and 12–17) has a lower odds of hospitalizations.

Similarly, female sex showed a minor likelihood of hospitalization in comparison with males. Several studies support this finding. A systematic review about 12 case series from China reported a proportion of male inpatients between 40% and 65% (Streng et al., 2020). Trends in case notification and hospitalization among symptomatic children aged 0–17 years in 10 European countries confirmed that male children were slightly more likely than female to be admitted in the hospital, reporting a higher crude attack rates for severe disease among males (Bundle et al., 2021; European Centre for Disease Control, 2021). By contrast, a broad systematic review and meta-analysis about studies from the first pandemic year found no association between sex and odds of severe disease or death (Harwood et al., 2022).

In northern Italy, ISTAT reports that education level are lower among foreign adults: 47.3% has at least a high school diploma *vs*. 64% of Italian adults (ISTAT, 2019, 2020). The education level seems to influence parents’ judgment on own children’ health status and potentially urgent conditions. Use of emergency care or specialist’s consultation are often the first choice also for non-urgent patients rather than family physicians, and this trend relates positively with lower education (Akbayram & Coskun, 2020). International studies found that less educated and poorer population groups have more often limited or inadequate health literacy (Hickey et al., 2018; Bonaccorsi et al., 2016). Related to this, recent findings highlight the impact of health literacy on health outcomes and behaviours. Low health literacy relates negatively with patient’s motivation, problem-solving ability, self-efficacy and disease knowledge. Indeed, during the COVID-19 pandemic both disease knowledge and health literacy significantly predicted preventive behaviours (Li & Liu, 2020). Moreover, health literacy effects the use of health care services, since children of parents with lower health literacy may be at higher risk of hospitalization and less use of services in the community (Adams et al., 2009; Berkman et al., 2011). In accordance, a systematic review found evidence to support that parents seeking care for their children at the Emergency Department have low health literacy, especially in case of children suffering of chronic disease as asthma (Morrison et al., 2013). Besides suggestions mentioned above, additional reasons could explain our results. Italy’s National Health Service guarantees equal healthcare levels free of charge to all children regardless of nationality, of their ethnic origin, their social or economic situation. A paediatrician, and whenever necessary, paediatric out- and in-patient care in one of the densely spaced hospitals, completely free of charge and equal for all are guaranteed for every child. Thus, equal access to healthcare no linked to distance from a hospital, family income and insurance status, excludes barriers to hospitalizations.

### Limitations

Limitations of this study include that it was conducted at a single region and it is limited to hospitalized patients, and may not be representative of children with COVID-19 in the ambulatory setting. In addition, data gathering is time-limited and concerns the initial period of pandemic during which safe and preventive measures consisted only in interpersonal contact restrictions and mask wearing. Hence, no evaluation about fluctuation of infections among children and adolescents related to vaccine introduction and virus mutated Variants of Concern (VoC) can be assessed. Since the European Medicine Agency (EMA) granted an extension for vaccination against SARS-CoV-2 in children aged 12 years onward in May 2021 (European Medicines Agency, 2021a), vaccination status of children cannot be considered for the study period. Even later, vaccine approval for children aged from 5 to 11 years occurred in November 2021 (European Medicines Agency, 2021b). Furthermore, spread of VoC (as Delta variant, B.1.617.2) causing infection escalation among paediatric population was reported in Italy after the study period (Public Health England, 2021).

The strength of this study is that it is the first large population-based report to demonstrate predictors on SARS-CoV-2 infection-related hospitalizations in children and adolescent.

## Conclusions

The current study reports additional data about epidemiology of COVID-19 among children and adolescents in Northern Italy, focusing on patients requiring tertiary care assistance. It shows a comparable trend of paediatric hospital admissions for COVID-19 and of the entire population hospitalizations over three waves. COVID-19 hospital admissions increase with a bimodal age distribution and the most admissions were among patients aged ≤4 and 5–11 years.

According to our results, male-sex, nationality and 0–4 age are predictors of hospitalization.

This study adds further information on epidemiology of COVID-19 among paediatric patients for the assessment and the implementation of efficient public health programs through targeted policy decisions.

## Supplemental Information

10.7717/peerj.15492/supp-1 Supplemental Information 1 Aggregated data.

## Additional Information and Declarations

### Data Availability

The following information was supplied regarding data availability: The raw data is available in the Supplemental File.
